# *Irf6* directs glandular lineage differentiation of epidermal progenitors and promotes limited sweat gland regeneration in a mouse burn model

**DOI:** 10.1186/s13287-018-0929-7

**Published:** 2018-07-04

**Authors:** Bin Yao, Wei Song, Zhao Li, Tian Hu, Rui Wang, Yihui Wang, Sha Huang, Xiaobing Fu

**Affiliations:** 1 Key Laboratory of Tissue Repair and Regeneration of PLA, and Beijing Key Research Laboratory of Skin Injury, Repair and Regeneration, First Hospital Affiliated to General Hospital of PLA, 51 Fu Cheng Road, Beijing, 100048 People’s Republic of China; 2 School of Medicine, Nankai University, Tianjin, 300052 People’s Republic of China; 3 Wound Healing and Cell Biology Laboratory, Institute of Basic Medical Sciences, General Hospital of PLA, Beijing, 100853 People’s Republic of China; 4 Tianjin Medical University, Tianjin, 300070 People’s Republic of China

**Keywords:** IRF6, Inductive SGC, SG regeneration, Burn environment

## Abstract

**Background:**

Damaged or malfunctioning sweat glands (SGs) after a burn injury would cause significant hyperthermia and even death, and there is an unmet need for effective treatment. Genetically reprogrammed stem cells show their potential advantages for inducing SG repair and regeneration.

**Methods:**

The expression of interferon regulatory factor 6 (IRF6) in skin was tested by immunofluorescence, and *Irf6* was overexpressed in epidermal progenitors (EPs) to stimulate SG differentiation. For in-vivo studies, second- and third-degree mouse burn wounds were treated with subcutaneous injection of EPs and *Irf6*-transfected cells, and cell retention and therapeutic effects were assessed.

**Results:**

IRF6 demonstrated differential expression between the footpad and dorsal skin and was upregulated along with embryonic and postnatal SG development. The *Irf6*-transfected cells converted their cell phenotypes as seen by gene and protein expression analyses and their morphology closely resembled epidermal-derived glandular cells. Inductive SG cell (SGC) transplantation and in-vivo tracing examination demonstrated that they could survive at damaged sites for 14 days. In comparison, the positive effects of inductive SGCs only result in restoring SG function in second-degree burn wounds but not in third-degree burn wounds as assessed by both perspiration tests and morphological analyses.

**Conclusions:**

These results suggest that IRF6 plays an important role in directing glandular lineage differentiation of Eps, but that the therapeutic efficacy of inductive SGCs may be restricted to the burn environment.

## Background

Eccrine sweat glands (SGs) are present all over human skin and have the important function of regulating body temperature. Improper thermoregulation can result in hyperthermia which could potentially lead to death [1]. A severe burn with overall skin architectural disruption poses a challenge for SG restitution during wound healing, requiring innovative strategies to promote tissue and functional repair. Stem cells are particularly attractive options, either by differentiating into SG cell types from epidermal-derived cells or indirectly from other germ layer-derived stem cells. Due to the plasticity, genetic reprogramming seems to be an alternative option; this relies on the appropriate and accurate transcription factors to modulate cell fate or induce cell differentiation [2].

Interferon regulatory factor 6 (*Irf6*) is a transcription factor characterized by a highly conserved pentatryptophan DNA binding domain and a less well-conserved protein interaction domain [3]. Mutations in *Irf6* cause two allelic orofacial clefting syndromes in humans: Van der Woude syndrome (VWS) and popliteal pterygium syndromes (PPS) [4]. *Irf6*-deficient mice have abnormal skin, limb, and craniofacial development [5, 6]. These earlier studies supported that *Irf6* plays an important role in epidermal development. However, whether *Irf6* is involved in glandular lineage differentiation of epidermal progenitors (EPs) is currently unknown.

On the other hand, tissue-specific microenvironments constitute a basic unit of physiology which integrate signals relayed to cells of the niche for interpretation [7–9]. The expression pattern of IRF6 in mice is in accordance with SG distribution. This shows that IRF6 is expressed more in footpads and is upregulated along with embryonic and postnatal SG development, which further supports their inductive potential. Hence, we hypothesized that transfecting *Irf6* in mouse EPs would direct the glandular lineage differentiation and further promote SG regeneration in vivo. Results showed that *Irf6* can direct the SG specification of EPs. The therapeutic effects of these inductive SG cells (SGCs) were then assessed using both second- and third-degree burn models in mice using perspiration tests and morphological analyses, which might help unravel the possible mechanisms underlying the observed SG regeneration.

## Methods

All animal procedures were performed in accordance with the guidelines of the Institutional Animal Care and Use Committee of the Chinese PLA General Hospital (Beijing, China).

### Animals

C57BL/6-Tg (ACTB-EGFP) 1Osb/J mice were purchased from Jackson Laboratories. Embryonic day 17.5 (E17.5), postpartum day 5 (P5), and postpartum day 28 (P28) C57BL/6 mice were obtained from HFK Bioscience Co., Ltd. (Beijing, China).

### Epidermal progenitor isolation

The back skin of newborn mice was cut and digested in trypsin at 4 °C overnight. The dermis was then adhered to the culture dish and carefully separated from the epidermis. The epidermis was diced into paste and digested with collagenase II (2 mg/ml) for 45 min with shaking every 10 min, and the digested products were filtered with a 40-μm cell strainer (Corning, USA) and centrifuged at 1000 rpm for 5 min to collect cells. The cells were cultured with EpiGRO™ human epidermal keratinocyte basal medium (Millipore, USA).

### Tissue immunofluorescence staining

All tissue sections were fixed in 10% formalin. After blocking and permeabilization with 0.5% Triton X-100% and 3% goat serum, cells were incubated with primary antibodies at 4 °C overnight, washed twice in phosphate-buffered saline (PBS) for 5 min, and incubated in Alexa Fluor 488-labeled secondary antibodies. 4′6-Diamidino-2-phenylindole (DAPI) fluoromount-G (Southern Biotech, USA) was used as a nuclear stain. Images were scanned with a confocal microscope (Leica, TCSSP8, Germany). Antibodies and dilutions used were as follows: K17 (rabbit, 1:200, Abcam), K8 (rabbit, 1:200, Abcam), K14 (rabbit, 1:200, Abcam), K18 (mouse, 1:200, Abcam), IRF6 (Rabbit, 1:200, CST), goat anti-rabbit Alexa Flour 488 (1:300, Beyotime, A0423), and goat anti-mouse Alexa Flour 488 (1:300, Beyotime, A0428).

### Retroviral infection

Plat-E cells were seeded at 8 × 10^6^ cells per 100-mm dish and incubated overnight. The next day, cells were transfected with pMXs vectors with Irf6 plasmid using X-tremeGene 9 (Roche). At 48 h after transfection, the virus-containing supernatants were collected, mixed, and used for transfection with 5 mg/ml polybrene. After 24 h the medium was replaced with complete F12 medium.

### Transition rate assay

Cell numbers were calculated through differential digestion because of the different adhesive capability between SGCs and EPs. First, transfected cells were trypsinized for 1 min and washed with PBS twice to discard parts of cells that were considered as untransformed, The cells were then digested with trypsin for another 3 min, centrifuged at 1000 rpm for 5 min, and cells that differentiated to SGCs were collected for counting. For the control group, cells were trypsinized for 1 min and collected for counting. The transfected cell numbers vs control is given as a transitional rate.

### Cell proliferation assay

Cell proliferation was evaluated through CCK-8 assay. Briefly, cells were seeded in 96-well plates at the appropriate concentration and cultured at 37 °C in an incubator for 4 h. When cells were adhered, 10 μl CCK-8 working buffer was added into the 96-well plates and incubated at 37 °C for 1 h. Absorbance at 450 nm was measured with a Tecan Infinite M200 Pro microplate reader.

### Quantitative real-time polymerase chain reaction (PCR)

Cells were lysed in TRIzol (Invitrogen) and RNA was isolated following the manufacturing protocol of the TRIzol reagent. Briefly, 200 μl chloroform was added per 1 ml TRIzol, then shaken for 15 s, incubated for 3 min at room temperature, and centrifuged at 12,000 rpm for 15 min at 4 °C. The aqueous phase containing the RNA was carefully transferred to a new tube and 0.5 ml isopropanol was added to the aqueous phase per 1 ml TRIzol. This was then incubated for 10 min at room temperature and centrifuged at 12,000 rpm for 15 min at 4 °C. The RNA precipitate formed a white pellet at the bottom of the tube. Total RNA was then reverse transcribed with the SuperScript VILO cDNA Synthesis Kit and amplified with the TaqMan™ Sample-to-SNP™ Kit (Invitrogen). All primers used were as per our previous study [10]. The PCR was carried out with a QuantStudio 5 Real-Time PCR Systems (Thermo Fisher Scientific) using the following procedure: initiation for 15 min at 95 °C, followed by 40 thermal cycles each at 95 °C for 10 s and 60 °C for 30 s, and then dissociation analysis. All data were analyzed with the C(t) value comparison method.

### Cell immunofluorescence staining

The cells were fixed in 4% paraformaldehyde (PFA) in PBS. Then the other procedure was carried out as previously described for tissue immunofluorescence staining.

### Burn model

To establish a paw pad burn model, mice were anesthetized with pentobarbital (100 mg/kg) and received preoperative subcutaneous buprenorphine (0.1 mg/kg). Second-degree and third-degree burns were administered to back paw pads with a soldering station (Weller, Germany) containing a temperature controlled round iron column. The size of the burn was a circle with diameter of 1 cm and the paw pad was treated with a working voltage of 300 V for 1 s for the second-degree burn and 3 s for the third-degree burn. Mice recovered in clean cages with paper bedding to prevent irritation or infection.

### Cell treatment

The green fluorescent protein (GFP)-labeled EPs and inductive SGCs were collected when cell confluency was 85–90% and injected into the paw pad of burn mice with Microliter™ Syringes (Hamilton, 7655–01), where one paw pad was injected with EPs and the other with inductive SGCs. The wild-type mice were killed 28 days later, and their feet were cut and fixed with 10% formalin overnight for paraffin section.

### Bioluminescence imaging

To track GFP-labeled cells in vivo, mice were anesthetized with pentobarbital (100 mg/kg) and received preoperative subcutaneous buprenorphine (0.1 mg/kg). They were then fixed with surgical tape to the working stage and faced to the light source. Bioluminescence imaging was performed to track the retention and the viability of cells on days 1, 3, 5, 7, 14, and 28 after injection using a Bruker image station system FX PRO (Bruker). High-sensitivity bioluminescent imaging was quantified by creation of polygonal regions of interest (ROIs) using the molecular imaging 7.1.1 software (Bruker).

### Hematoxylin and eosin (H&E) staining

The samples were fixed with 10% buffered formalin and processed for paraffin embedding. Sections were cut to a thickness of approximately 5 μm and placed on glass slides. The sample sections were routinely stained with H&E for morphological assessment with a microscope (Olympus, CX51, Japan).

### Sweat test

For the iodine sweat test, the hind paws of the mice were painted with 2% iodine/alcohol (wt/vol). Once dry, the paws were covered with 40% starch/oil suspension (wt/vol). Dark spots revealed functional sweat pores. The numbers of sweat dots on the mouse paws were counted by multiple investigators in a blinded manner and each group repeated with at least three mice.

## Results

### Immunofluorescent staining of IRF6 at tissue level of mouse dorsal and plantar skin

**Fig. 1:**
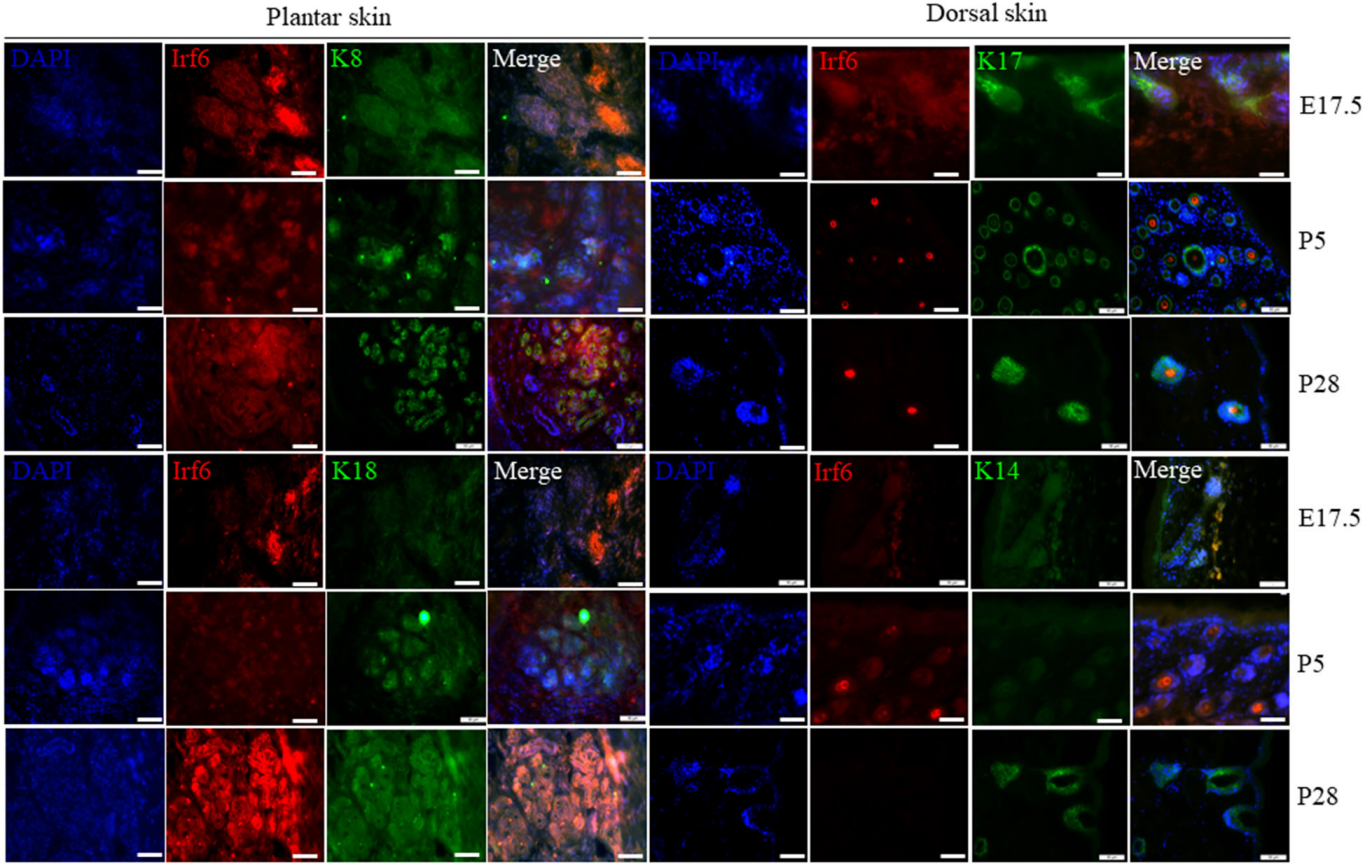
Differential expression of interferon regulatory factor 6 (Irf6) in dorsal skin and plantar skin of mice. The hair follicle-specific marker cytokeratin (K)17 and epidermal-specific marker K14 were coexpressed in dorsal skin. The sweat gland-specific markers K8 and K18 were coexpressed in plantar skin (K14, K17, K8, K18: green; IRF6: red; cell nucleus: blue). DAPI 4′6-diamidino-2-phenylindole, E embryonic day, P postpartum day

In mice, SG germ cells emerge at embryonic day 17.5 (E17.5) and during postpartum days 1–5 (P1–P5), and SG ducts extend deeply into the dermis and form a coiled gland at the tip at P5; the SG completely mature at P21 and glands become fully functional at P28. To verify the role of *Irf6* in SG development, we tested its expression at the key points of E17.5, P5, and P28. It has been found that *Irf6* is a critical transcriptional regulator of epidermal differentiation [4–6]. More specifically, *Irf6* is expressed in the suprabasal epidermis at E17.5, the developmental point at which SG placodes emerge. In this regard, we examined the expression of IRF6 during the developmental process [11]. Immunofluorescence studies in a series of footpads and dorsal skin were obtained. As shown in Fig. 1, IRF6 coexpressed with the hair follicle marker cytokeratin (K)17 and EP marker K14 in back skin only at E17.5, but coexpressed with the SG markers K8 and K18 in the paw pad during SG development. In addition, IRF6 expression in back skin was similar with plantar at E17.5, while expression of IRF6 in plantar skin was increased following the SG development but decreased rapidly in dorsal skin.

### *Irf6*-induced EPs exhibit SGC properties

**Fig. 2:**
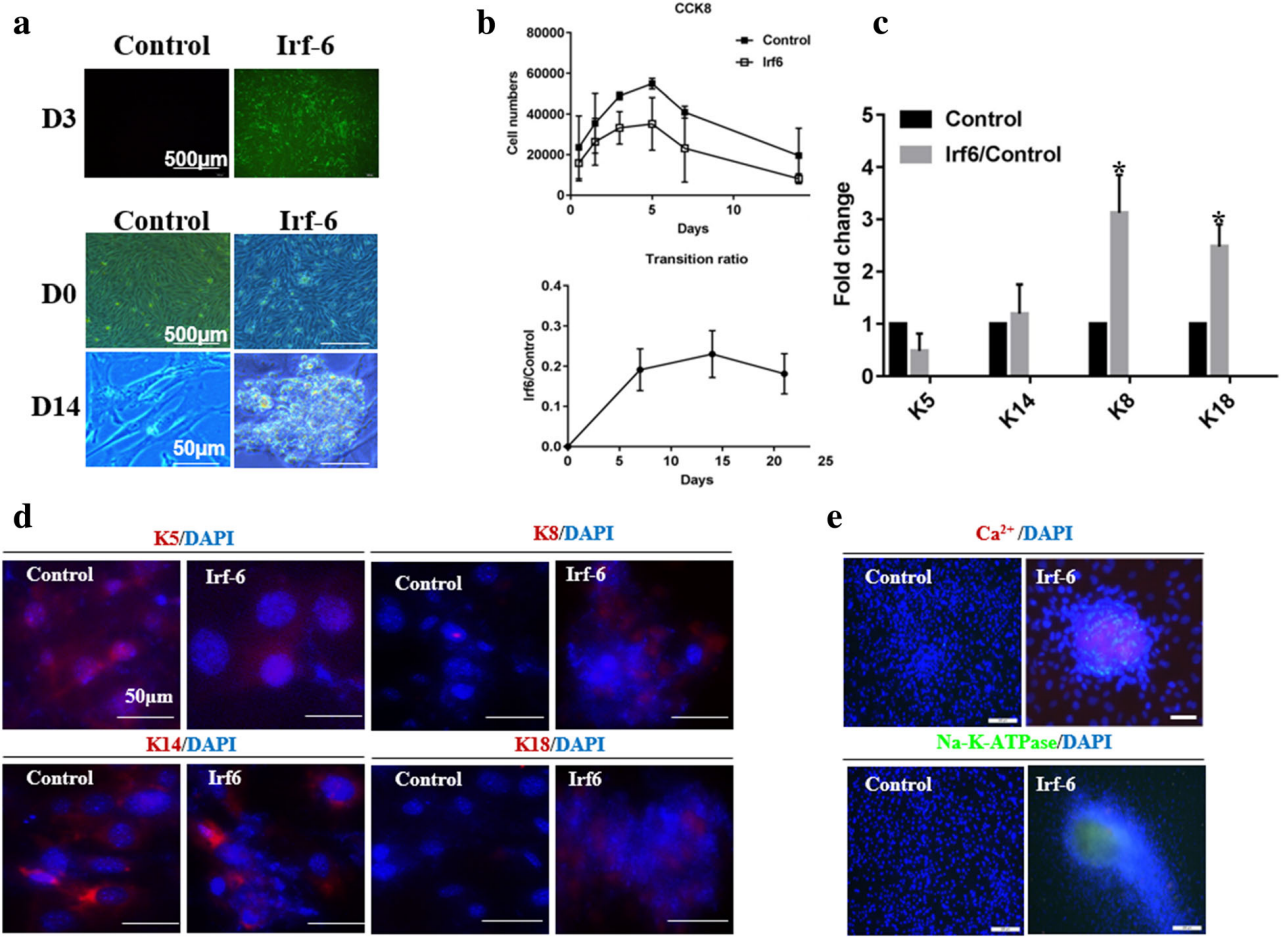
Sweat gland characteristics were observed in transfected cells. **a** Transfection efficiency at day (D)3 and morphological changes of transfected cells and control. **b** Proliferative capacity and transition ratio of transfected cells. **c** Transcriptional level of epidermal- and sweat gland-specific markers were detected by quantitative PCR. **d** Translational levels of specific markers were detected by immunofluorescence staining. **e** Sweat gland function was detected by Ca^2+^ and Na-K-ATPase. DAPI 4′6-diamidino-2-phenylindole, Irf6 interferon regulatory factor 6, K cytokeratin

To verify the potential of *Irf6* for SG differentiation, we transfected *Irf6* into EPs and transfection efficiency was more than 90% after 48 h. These cells have undergone a transformation in cell morphology, becoming bright and with a clustered appearance after 14 days of induction (Fig. 2a). Interestingly, cells transfected with *Irf6* exhibited a stick-like morphology which was different from the ellipse appearance of native SGCs. At the same time, we examined the transitional rate and cell proliferation and found that the ratio of SG-like cells was increased with inductive duration of *Irf6* overexpression and the cell numbers were reduced relative to control (Fig. 2b).

Following infection, EPs were maintained for 14 days before RNA extraction or immunocytochemistry. For SGC and EP identification, we selected K5, K14, K8, and K18 as biomarkers according to our previous research [10]. As expected, during the whole developmental process the expression of K5, K14, K8, and K18 was increased in transfected cells (Fig. 2c). K8 and K18 were obviously upregulated in induced SGCs relative to scramble-infected EPs, whereas K5 expression was decreased. The expression of K14 did not show any significant difference (Fig. 2d). Moreover, the increase in free Ca^2+^ concentration and Na-K-ATPase expression revealed that *irf6* overexpression facilitates secretory function (Fig. 2e).

Taken together, these data indicate that *Irf6* has a strong effect on SGC marker mRNA, protein expression, and secretory function.

### Engraftment of transfected cells into the burn paw and in-vivo SG regeneration

**Fig. 3:**
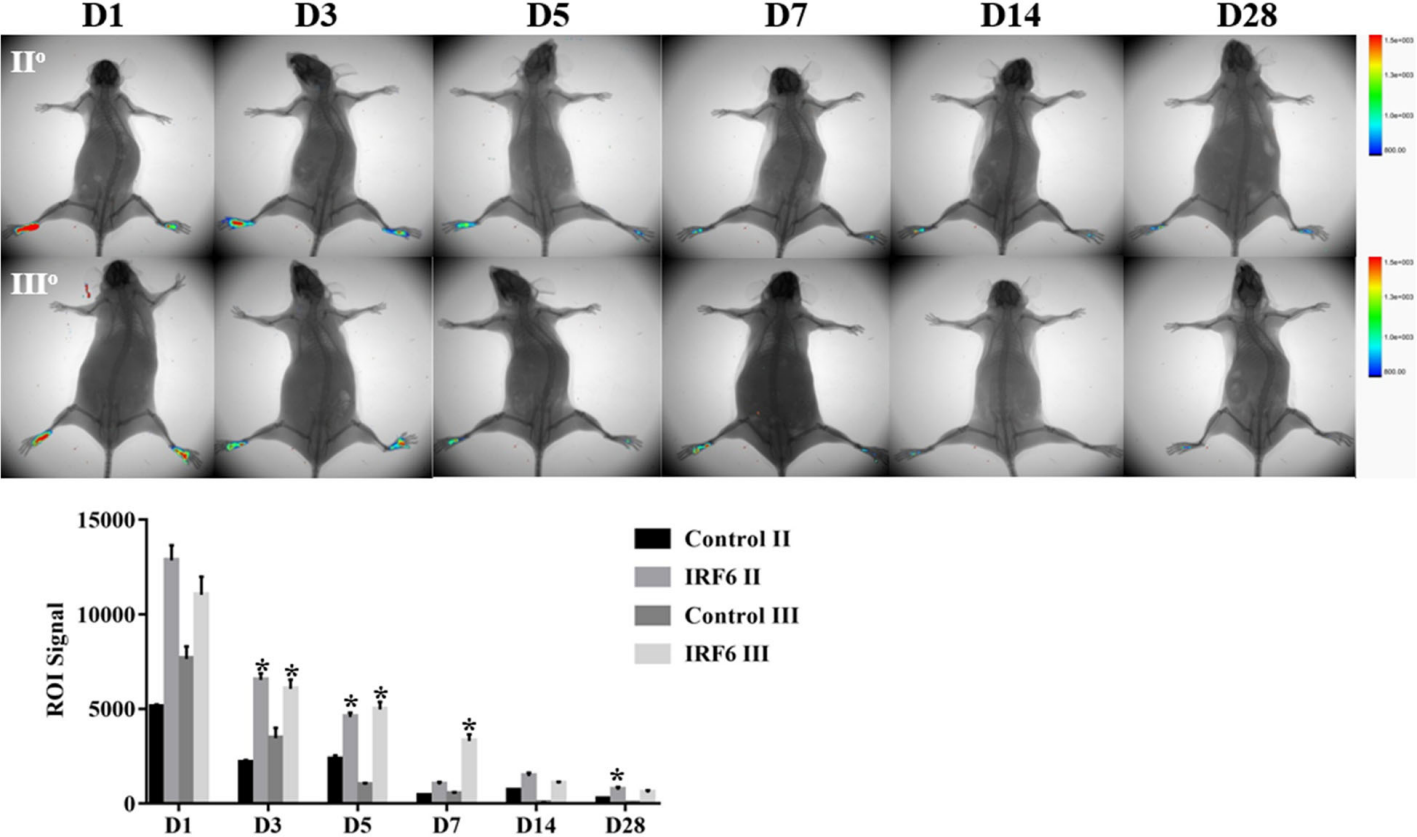
Cell retention and viability were detected by bioluminescence imaging. *p < 0.05. D day, Irf6 interferon regulatory factor 6, ROI region of interest

We next evaluated whether these inductive SGCs that were isolated through fluorescence-activated cell sorting with K18 antibody would contribute to the repair of wounded skin, especial SG regeneration in burn mice. To this end, GFP-labeled EPs and inductive SGCs were injected to second- and third-degree burn wounds. We first traced the cells through an in-vivo animal imaging system. The retention of inductive SGCs in the wound was far higher than for Eps, and the fluorescence signals decreased gradually after injection on days 1, 3, 5, 7, 14, and 28 both in second- and third-degree burn wounds, while there was a little more cell persistence in second-degree burn wounds (Fig. 3).

**Fig. 4:**
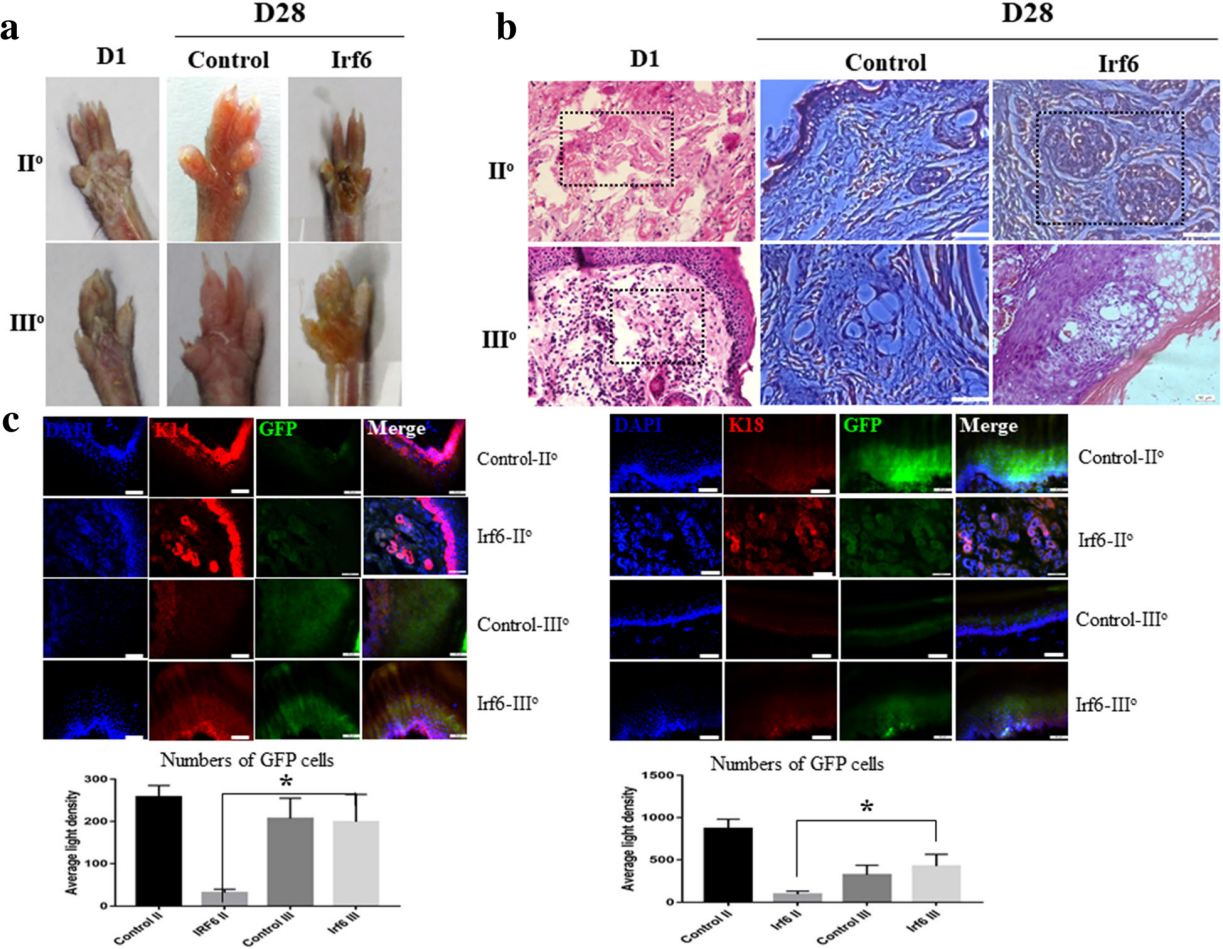
Morphological changes and protein expression of treated plantar tissue. **a** Sweat test in treated plantar paw and control. **b** Morphological changes were detected by H&E staining in treated plantar paw and control. **c** Protein expression and labeled-cell involvement in different levels of burn injury (cytokeratin (K)14 and K18: red; inductive cells: green; cell nucleus: blue). D day, DAPI 4′6-diamidino-2-phenylindole, GFP green fluorescent protein, Irf6 interferon regulatory factor 6

We performed perspiration tests on the burned paws of mice based on iodine/starch at day 28 after transfected cell injection. Only mice with second-degree burns showed individual SGs (black dots) and the number increased within 10 min; however, no obvious black dots were observed in the paw pad of control and third-degree burn mice (Fig. 4a). Histological analysis showed that a second-degree burn only destroyed the original SGCs but kept the supporting structure of the SG, while a third-degree burn destroyed both the original SGCs and the supporting structure. At 28 days after treatment, SG regeneration was only observed in second-degree burn wounds by inductive SGC transplantation (Fig. 4b).

Immunofluorescence staining confirmed the regenerative capability of inductive SGCs for wound repair. However, inductive SGCs only contributed to SG regeneration in second-degree burn wounds (K18-positive expression) while, in third-degree burn wounds, cells were involved in epidermis repair (K14-positive expression) but not in SG regeneration (Fig. 4c). Notably, the number of GFP-positive cells did not show significant difference between the EP-treated groups with third-degree burns and second-degree burns; however, they were more GFP-positive cells in third-degree burns compared with second-degree burns in the SGC-treated group, which suggested that SGCs promoted epidermis repair more strongly than SG.

## Discussion

Mammals need to precisely regulate their core body temperature since tissues and organs, specifically the brain, are vulnerable to overheating [12]. Extraordinary number of eccrine glands allows humans to eliminate excess heat very efficiently and patients who lose the sweating function are in the danger of heat stroke, or even death [11]. Although regeneration may be therapeutically achieved by endogenous regeneration, SG progenitors are limited by a poor regenerative capability and vulnerability to the neighboring niche which is often damaged by the wound [13]. Therefore, there is an urgent need to develop innovative and effective strategies for SG regeneration. Over the last decade, the transplantation of exogenous reprogrammed cells has become capable of restoring the function of various critical organs [14, 15]. Unfortunately, to our knowledge, no “master regulator” of SG differentiation, such as myoblast determination protein (MyoD) in skeletal muscle [16], has been identified.

*Irf6* has been reported to function as a critical regulator of proliferation and differentiation in keratinocytes and is essential for epidermis development. In this study, we found the expression of IRF6 in plantar skin was in accord with SG development at the bud, neonatal, and adult stages (E17.5, P5, and P28). Expression started at the initial stage and increased with sweat duct formation and the secretory portion maturation, which suggested that IRF6 might be involved in induction of SG germ formation, down-growth, and secretory coil formation. Therefore, we assumed that *Irf6* may be one of the master regulators of SG cell fate determination.

In this study, the *Irf6*-transfected cells spread faster and aggregated as clusters without a specific shape. They also expressed K8 and K18, the specific genes of the secretory portion, demonstrating that *Irf6* could drive glandular differentiation. Interestingly, although K5 expression of the *Irf6*-transfected cells was decreased, K14 expression did not reduce. This suggested that some of the transfected cells might differentiate into myoepithelial cells.

An in-vivo assay showed that the inductive SGCs could promote SG regeneration and repair SG function in second-degree burn wounds. However, the therapeutic efficacy of inductive SGCs on third-degree burn wounds was limited. This situation may be explained, at least in part, by the different burn environments involved. In second-degree burn wounds, the original SGCs were destroyed but the supporting structure of the SG remained intact, while in third-degree burn wounds both the original SGCs and the supporting structure were destroyed. H&E stained images showed that the impaired regions were filled with newly migrated cells, which may be transplanted cells or activated endogenous stem cells. Furthermore, GFP-labeled cell tracing showed that transplanted cells were involved in functional SG repair regeneration in second-degree burn wounds but epidermis repair in third-degree burn wounds.

These results strongly correspond with our immunofluorescence analysis of specific SG protein expression. Given the different traits between second- and third-degree burn wounds, we hypothesized that environmental signals play an indispensable role in the therapeutic efficacy of inductive SGCs in burn mice. Although these results are shown in the present study, the effects of inductive SGCs on SG regeneration were only examined in burn wounds. Future studies, with different methods according to the different kinds of wound, are required to better determine if environmental signals of wounds lead to the limited therapeutic efficacy of inductive SGCs.

## Conclusions

In summary, we have provided evidence that IRF6 expression along with SG development and *Irf6* is sufficient to direct the SG specification of EPs in vitro. In addition, the inductive SGCs could repair SG both structurally and functionally in second-degree burn wounds, but they had limited regenerative capability in three-degree burn wounds. These results confirmed previously unreported roles of Irf6 in SG development and differentiation, and indicated that the inductive cell and its related therapeutic efficacy during wound healing is more complex than initially thought. Although the detailed mechanisms of specific environmental signals remain to be identified, their indispensable role explains, in part, the different therapeutic efficacy exhibited in vivo.

## Abbreviations

DAPI: 4′6-Diamidino-2-phenylindole
EP: Epidermal progenitor
GFP: Green fluorescent protein
H&E: Hematoxylin and eosin
Irf6: Interferon regulatory factor 6
MyoD: Myoblast determination protein
PBS: Phosphate-buffered saline
PCR: Polymerase chain reaction
PPS: Popliteal pterygium syndromes
SGC: Sweat gland cell
SG: Sweat gland
VWS: Van der Woude syndrome
